# Teachers’ Well-Being and Health in Germany: Representative Cross-Sectional Study

**DOI:** 10.2196/94961

**Published:** 2026-06-04

**Authors:** Rebekka Schröder, Tim Hamer, Ralf Suhr, Lars König

**Affiliations:** 1 Stiftung Gesundheitswissen Berlin, Berlin Germany

**Keywords:** Germany, health literacy, health, representative, teachers, well-being

## Abstract

**Background:**

Teachers play a fundamental role in the educational system and carry significant responsibilities. However, various occupational demands may compromise their health and well-being. At present, empirical data on the specific correlates of health and well-being among teachers in Germany remain limited, for example, regarding the relevance of health literacy.

**Objective:**

The primary aim of this study was to conduct a comprehensive assessment of the current health and well-being status among teachers. Specifically, the study aimed to quantify these factors according to the Positive Emotions, Engagement, Relationships, Meaning, and Accomplishments (PERMA) model as well as their general and mental health literacy. Secondary aims were to explore how health and well-being are associated with general and mental health literacy, as well as other personal and professional factors.

**Methods:**

This cross-sectional web-based survey was conducted in 2022 among teachers currently working in Germany. Participants were randomly sampled from all teachers in a general population panel (>100,000 panelists recruited exclusively offline). In total, 61.6% (1005/1631) of the invited teachers participated (mean 49.99, SD 9.74 years). The final sample was weighted by gender, age, school type, and federal state. Health and well-being (PERMA Profiler questionnaire), general health literacy (European Health Literacy Survey Questionnaire, 16 items), mental health literacy (Mental Health Literacy Tool for the Workplace), sociodemographic (eg, gender and age), and professional information (school type, experience, and teaching load) were assessed. Bivariate (ANOVAs, *t* tests, and Pearson correlations) and multivariate (multiple regression) analyses were computed to analyze the associations between the health and well-being outcomes and the other factors.

**Results:**

The teachers reported intermediate to high well-being and health according to the PERMA model. In addition to significant bivariate associations, regression analysis for the well-being outcome (*F*_13, 977_=10.66; *P*<.001) revealed general (β=.23; *P*<.001) and mental health literacy (β=.15; *P*<.001) as significant positive predictors. In addition, teachers aged 40-49 years (β=–.12; *P*=.002) and 50-59 years (β=–.10; *P*=.03), as well as those with 10-19 years of experience (β=–.08; *P*=.04), reported significantly lower well-being than their younger/early-career peers. The regression model for the health outcome (*F*_13, 980_=8.34; *P*<.001) showed that general health literacy (β=.25; *P*<.001), teaching at nonprimary schools (β=.10; *P*=.002), and a high teaching load (≥28 hours: β=.10; *P*=.02) were positively associated with the health outcome. Teaching experience of 10-19 years (β=–.09; *P*=.02) and older age (50-59 years: β=–.12; *P*=.02; ≥60 years: β=–.09; *P*=.046) predicted lower health scores. Other factors were not significant in the well-being and health regression models (all *P*>.05).

**Conclusions:**

This study highlights the potential of health literacy as an intervention target for improving teachers’ health and well-being. To build on these findings, longitudinal and interventional studies are needed.

## Introduction

Teachers play a crucial role in shaping the development of future generations [1,2]. However, teachers are often faced with high workloads and stressful job demands. These include physical factors such as noise, as well as psychological demands such as the division of attention, intrusions of work into free time, and conflicting standards and expectations posed by students, parents, and superiors. These factors can affect teachers’ physical and mental health [3-5]. Therefore, it is not surprising to see in synthesized data from international publications that a significant proportion of teachers experience stress, anxiety, depression, and burnout [6]. In addition, burnout among teachers has been linked to various physical health problems [7]. In Germany, teachers experience psychological or psychosomatic disorders more often than individuals in other professions [3]. Moreover, specific symptoms such as exhaustion or stress-related factors can be observed more frequently in teachers than in other professions [3,5]. Many teachers also experience physical health issues such as elevated blood pressure [8]. These findings have prompted research focused on improving the understanding of the status, determinants, and facets of teachers’ well-being and health in an effort to reduce negative outcomes, promote healthy working conditions, and ultimately increase job satisfaction and retention [9-12].

Well-being can be defined, conceptualized, and operationalized in various ways [10,13]. According to a widely used model in positive psychology, well-being is conceptualized as a multidimensional construct comprising 5 elements that independently contribute to well-being: Positive Emotions, Engagement, Relationships, Meaning, and Accomplishments (PERMA) [14]. The concept of well-being according to the PERMA model thus goes beyond happiness as it not only encompasses temporary positive mood but is a broader and—at least in parts—both subjectively and objectively measurable construct that enables individuals to flourish [14].

Despite the numerous demands in teaching roles [3-5], many quantitative investigations report that teachers demonstrate medium to good overall well-being across different theoretical frameworks and conceptualizations [10,11]. Furthermore, a recent study explicitly studying the PERMA model among teachers in a German convenience sample showed positive associations between 3 of the 5 core components of PERMA (ie, positive emotion, relationships, and accomplishments) and job satisfaction [15]. However, this study solely focused on the PERMA components without quantifying the overarching well-being factor.

A number of different factors have been identified that influence teachers’ well-being and health [3,11,16]. These encompass solely personal factors (eg, gender and age), personal work-related factors (eg, type of school, teaching experience), and nonpersonal work-related factors (eg, school size and school funding). For example, it has been shown that contextual factors summarized under the umbrella term school climate, such as resources, feelings of belonging (eg, affiliation), autonomy, and teacher-student relationships, play an important role in addition to individual behavioral factors. One important aspect of the latter is the active shaping of processes, relationships, and attitudes in order to align personal factors with job demands [11,16].

Higher well-being of teachers might not only be a desirable goal in itself. It can, in turn, also have an impact on other outcomes and thus constitutes an integral element for promoting supportive student-teacher relationships, good classroom management, and ultimately a positive school environment to facilitate successful learning [9,17]. Specifically, outcomes influenced by teachers’ well-being have been observed at the individual level (such as sleep quality and intention to stay in the job), as well as in their teaching practices and, eventually, also in their students (such as student engagement and well-being [9,18]).

While existing literature has identified many determinants of teachers’ well-being and health, far less attention has been paid to the roles of teachers’ health-related competencies in shaping these outcomes. Health literacy has been identified as a key factor contributing to many health-related outcomes, including well-being [19-21]. Specifically, health literacy encompasses knowledge, motivation, and competencies of accessing, understanding, appraising, and applying health-related information regarding health care, disease prevention, and health promotion [21]. Beyond this general conceptualization of health literacy, more specific facets have been developed. Mental health literacy, in particular, has emerged as a critical field, applying the concept of health literacy to mental health. More specifically, it refers to understanding how to obtain and maintain mental health, understanding mental disorders and their treatments, reducing the associated stigma, and promoting help-seeking for mental health struggles, and is thus tightly interlinked with well-being [22]. In addition to the individual benefits of higher levels of health literacy, both mental and general health literacy have been identified as critical factors for health education and promotion in the educational system [23-27].

According to a 2017 nonrepresentative survey across 2 federal states in Germany, only about 50.1% of teachers demonstrated sufficient general health literacy [25]. In another study, this proportion was found to be notably higher among school leaders using a different questionnaire (70.7% [24]). Regarding teachers’ mental health literacy, limited research has explicitly evaluated the status quo, with existing literature predominantly focusing on interventions [27,28]. For example, one study used a single self-report item in a convenience sample and found a low to intermediate average level of mental health literacy among the teacher participants [29]. Other publications on health literacy in German teachers are limited to conference abstracts or focus on more specific facets of health literacy, for example, in the digital space or at an organizational level [30-33].

Despite its importance, few studies to date have explicitly studied how general and mental health literacy in teachers relate to their well-being, health, and other personal factors within the German educational system. Therefore, the aims of this study were twofold: first, we aimed to collect current data quantifying well-being and health according to the PERMA model, as well as levels of general and mental health literacy among teachers living and working in Germany. Second, we aimed to explore how health and well-being are associated with general and mental health literacy as well as with other personal and professional factors.

## Methods

### Ethical Considerations

A study protocol was reviewed by the ethics committee of the Berlin Medical Association. The committee did not raise any ethical or professional objections to the study protocol (reference Eth-39/22). Before data collection, all participants provided informed consent to take part in the study. The study was funded by the independent, nonprofit foundation Stiftung Gesundheitswissen. Data collection was conducted by a market research institute (forsa Gesellschaft für Sozialforschung und statistische Analysen mBH). The market research institute provided anonymized data to the Stiftung Gesundheitswissen. The participants did not receive any direct compensation from the foundation, and the foundation did not have any influence over the data collected or the nature of the results.

This study was part of a comprehensive research project that collected data on various topics and in multiple samples. Results are presented in separate publications with different thematic foci. Some parts of this project have already been published [34-36], and further publications will likely follow.

### Survey Methodology and Data Acquisition

Data for this study were collected by the market research institute forsa using their online panel forsa.omninet. This panel is representative of individuals in the general population in Germany who speak German, have internet access, and are aged 14 years and older. At the time of data collection, the panel had about 100,000 members. The composition of the panel is continuously monitored regarding key characteristics (eg, region, age, and gender). Recruitment is adjusted accordingly to ensure that all relevant segments of society are represented. The market research institute had information on whether the participants were teachers from prior surveys. A random sample was drawn from all teachers in the panel, and the selected individuals were invited via email. Before data collection, participants confirmed that they still work as teachers. There were no further inclusion or exclusion criteria. Data collection took place in September and October 2022. A total of 1631 teachers were invited to take part in the study, of which 1005 followed the invitation and completed the survey, corresponding to a response rate of 61.6%.

To reduce the potential risk of bias due to missing data from specific segments of the teacher population or overrepresentation of other segments, the market research institute computed survey weights using an iterative proportional fitting approach with the following weight variables and combinations: (1) gender × age (≤39 years, 40-49 years, 50-59 years, and ≥60 years), (2) type of school (referring to 7 types of schools: Grundschulen [primary schools], Hauptschulen [lower secondary schools], Realschulen [intermediate secondary schools], Schulen mit mehreren Bildungsgängen [schools with multiple educational tracks], Gymnasien [higher secondary schools to obtain a university entrance qualification], Förderschulen [special needs schools], and andere Schulformen [other schools; in German due to the specificity of the German educational system), and (3) federal state. Weighting was informed by the school statistic update of the German Federal Statistical Office (German: Schulstatistik für allgemeinbildende Schulen des statistischen Bundesamts, 2021/2022). The weighting procedure assigns a single weighting factor to each individual that is then applied in the statistical analyses.

### Measures

#### Overview

Data were collected online using web-based surveys. Participants could choose not to answer any item, resulting in missing values for some questions. Sum scores or mean values were not calculated for any scales that had missing items for that participant.

#### Sociodemographic Information

Participants provided basic sociodemographic information, including gender (male or female) and age. They were then sorted into 4 age groups (≤39 years, 40-49 years, 50-59 years, and ≥60 years) for further analyses.

#### Professional Factors

The participants were asked to indicate the type of school they currently teach at. To ensure adequate statistical power given the federal state-specific nature of the German educational system, these responses were dichotomized into 2 groups for analysis: primary school teachers and those teaching at all other school types.

In addition, participants were asked to indicate for how long they have been working as a teacher since finishing their professional education (German: Referendariat): 1-9 years, 10-19 years, or ≥20 years. Lastly, they reported the number of lessons they teach in an average week, that is, their teaching load (German: Deputat), with the following response options: <15 lessons, 15-19 lessons, 20-24 lessons, 25-27 lessons, and ≥28 lessons.

#### PERMA Well-Being and Health Within the PERMA Framework

The German version of the PERMA Profiler questionnaire was used to assess overall well-being and health [37,38]. The PERMA Profiler instrument assesses well-being according to the PERMA model as a multidimensional construct based on 5 core components (Positive Emotions, Engagement, Relationships, Meaning, and Accomplishments). The questionnaire consists of 23 items. There are 3 items for each of the 5 PERMA components. Two additional 3-item scales refer to Negative Emotions and Health. Moreover, there is 1 item referring to Happiness and 1 item referring to Loneliness. The 8 additional items beyond the core concept of PERMA well-being act as filler items to disrupt response tendencies and provide additional information to provide a more comprehensive insight into the participants’ mental health. Of the 23 items, 10 items are rated on 11-point Likert scale ranging from “never” (0) to “always” (10), 11 items are rated on a 11-point Likert scale ranging from “not at all” (0) to “completely” (10), and 2 items are rated on an 11-point Likert scale ranging from “terrible” (0) to “excellent” (10). For this investigation, an overall score of PERMA well-being is calculated as the mean of all 15 PERMA core items (ie, all items referring to Positive Emotions, Engagement, Relationships, Meaning, and Accomplishments). In addition, the mean value for the health subscale was calculated as the average value across the 3 health items. Higher values in both the well-being score and the health score denote higher expression of the construct. As the PERMA Profiler was primarily developed to assess well-being [38], not health, we added a brief analysis of convergent validity for the health scale. Specifically, we computed the Pearson correlation between the mean PERMA health scale value and an additional single item assessing physical health (“Overall, how would you rate your physical health at present?”). Responses of the single item were recorded on a scale ranging from “very bad” (0) to “very good” (10).

#### General Health Literacy

The German translation of the 16-item version of the European Health Literacy Survey Questionnaire (HLS-EU-Q16 [39-41]) was used to assess levels of general health literacy of the participants. This questionnaire asks the participants to indicate their subjective difficulty in accessing, understanding, appraising, and applying health information concerning health care, disease prevention, and health promotion. Each of the 16 items is answered on a 4-point Likert scale (“very easy,” “fairly easy,” “fairly difficult,” and “very difficult”). To obtain an overall score, the individual item responses are dichotomized in a first step (assigning a value of 1 to “fairly easy” and “very easy” responses and a value of 0 to “fairly difficult” and “very difficult” responses). Then, a sum score is calculated by adding up these values across the dichotomized items. The resulting sum score has a range of 0-16, where higher values refer to higher levels of general health literacy. Cutoff scores were established to classify individuals according to their individual sum scores into the following groups: inadequate and problematic health literacy (scores 0-12), and adequate health literacy (scores 13-16 [42]).

#### Mental Health Literacy

An adapted form of the German version of the Mental Health Literacy Tool for the Workplace (MHL-W-G [43,44]) questionnaire was used to assess mental health literacy. The instructions were slightly modified by adding the notion that participants should imagine they were currently working, if that was not actually the case. However, as all participants in this sample were currently working as teachers, this addition was not relevant to them. The adaptation was only made to ensure that results were comparable to samples that also include unemployed participants [34]. The questionnaire consists of 4 sets of 4 questions. Each set of questions is presented alongside a case vignette. In each vignette, a short portrayal of a coworker who experiences mental health issues is given, including a description of the person’s current mental state, symptoms, and life circumstances. Two case vignettes refer to female coworkers, and 2 case vignettes refer to male coworkers. In addition, the vignettes differ in the nature of the mental health problem without explicitly labeling it. For each vignette, the participants answer the same set of 4 items. These refer to the ability to recognize specific mental disorders, knowledge and beliefs about risk factors and prevention, knowledge and attitudes to facilitate help-seeking, and knowledge and beliefs about mental health interventions. Specifically, the participants were asked to rate their knowledge about what might be happening, how they could prevent the situation from worsening, what to say or do in the situation, and which resources and services might be helpful on a 5-point Likert scale with the poles “disagree” (1) and “agree” (5) in the German translation. The ratings were combined into a sum score across all items and vignettes. This sum score ranges from 16 to 80, with higher values indicating higher mental health literacy. So far, no cutoffs to categorize individuals with low and high levels of mental health literacy according to this questionnaire have been established, which is why the sum score is analyzed as a continuous variable.

### Statistical Analyses

All statistical analyses were conducted with IBM SPSS Statistics (version 29.0.2.0) on the weighted dataset (details on weighting can be found above in the “Survey Methodology and Data Acquisition” section). A significance level of α=.05 was used for all inferential analyses. Data analysis proceeded in 3 steps: first, to ensure quality, Cronbach α was computed as a measure of the internal consistency of all multi-item questionnaire scales, and interpreted according to the following rule of thumb: >0.90 excellent, >0.80 good, >0.70 acceptable, >0.60 questionable, >0.50 poor and <0.50 unacceptable [45]. Convergent validity of the PERMA health scale was evaluated by computing a Pearson correlation between the average scale scores and the single-item health indicator. Second, bivariate associations between the independent variables and PERMA well-being and overall health according to the PERMA scale were evaluated using Pearson correlations (continuous factors), independent 2-tailed *t* tests (dichotomous factors), and ANOVAs (multilevel factors). Pearson correlation coefficients are reported alongside their 95% CIs. If ANOVAs resulted in significant main effects, additional pairwise *t* tests were computed to test differences between each pair of factor levels using a Bonferroni-corrected α level. For these post hoc tests, uncorrected *P* values are reported along with the corrected α-thresholds. Effect sizes are reported as Cohen *d* for *t* tests and η^2^ for ANOVAs, with small, medium, and large effects defined as 0.20, 0.50, and 0.80 for *d* and 0.01, 0.06, and 0.14 for η^2^ [46]. Degrees of freedom were adjusted in all *t* tests where the Levene test for equality of variances indicated that variances were not homogeneous. Normality assumptions for these analyses were not formally tested, given the robustness of these analyses even in nonnormal data distributions [47,48]. Third, 2 multiple linear regression analyses (forced entry method) were conducted to identify the unique predictive value of each variable for both PERMA well-being and health. For these analyses, general and mental health literacy were entered as continuous predictors. Gender, age, type of school, teaching load, and experience were dummy-coded and analyzed as additional dichotomous predictors.

## Results

### Sample Characteristics and Descriptive Statistics

The sample consisted of 1005 individuals, with an average age of 49.99 (SD 9.74) years. Detailed characteristics of the sample before and after the weighting process can be found in Table 1.

**Table 1:** Characteristics of the sample before and after the weighting process. Cumulative percentages may exceed or fall below 100% due to weighting and rounding.

Variable							Unweighted sample, n (%)	Weighted sample, n (%)
**Gender**								
					Male		356 (35.4)	272 (27.1)
					Female		649 (64.6)	733 (72.9)
**Age groups**								
						≤39 years	178 (17.7)	368 (36.6)
						40-49 years	266 (26.5)	268 (26.6)
						50-59 years	368 (36.6)	259 (25.8)
						≥60 years	193 (19.2)	110 (10.9)
**Type of school**								
		Primary school					228 (22.7)	300 (29.8)
		All other types of school					777 (77.3)	705 (70.2)
**Teaching load**								
	<15 lessons						122 (12.1)	124 (12.4)
	15-19 lessons						131 (13.0)	145 (14.4)
	20-24 lessons						263 (26.2)	250 (24.9)
	25-27 lessons						369 (36.7)	335 (33.3)
	≥28 lessons						119 (11.8)	151 (15.0)
		Missing					1 (0.1)	1 (0.1)
**Years professionally teaching**								
				1-9 years			193 (19.2)	317 (31.5)
				10-19 years			309 (30.7)	339 (33.7)
				≥20 years			499 (49.7)	343 (34.2)
				Missing			4 (0.4)	6 (0.6)
**Health literacy**								
			Inadequate/problematic				248 (24.7)	265 (26.4)
			Adequate				755 (75.1)	738 (73.4)
			Missing				2 (0.2)	2 (0.2)

### Quality of Measures

Internal consistency of the questionnaire scales was excellent for the PERMA health scale, good for the PERMA well-being total score and the MHL-W-G total score, and acceptable for the HLS-EU-Q16 total score. Detailed information can be found in Table 2.

Underlining the convergent validity of the PERMA health scale, responses on that scale correlated positively with the single physical health item (*r*=0.77, 95% CI 0.74-0.79; *P*<.001).

**Table 2:** Cronbach α indices of internal consistency and the number of items in the survey questionnaires.

Scale	Cronbach α	Items, n
PERMA^a^ well-being	0.89	15
PERMA health	0.91	3
HLS-EU-Q16^b^	0.78	16
MHL-W-G^c^	0.88	16

^a^PERMA: Positive Emotions, Engagement, Relationships, Meaning, and Accomplishments.

^b^HLS-EU-Q16: European Health Literacy Survey Questionnaire, 16 items.

^c^MHL-W-G: German version of the Mental Health Literacy Tool for the Workplace.

### Descriptive Findings

Detailed descriptive statistics of the PERMA, HLS-EU-Q16, and MHL-W-G scales can be found in Table 3. Overall, participants reported intermediate to high well-being and health (according to the PERMA model) relative to the range of the questionnaire.

**Table 3:** Descriptive statistics of the survey questionnaires across the entire sample.

	n	Mean (SD)	Minimum-maximum
PERMA^a^ well-being	1002	7.18 (1.18)	1.47-9.47
PERMA health	1005	6.64 (1.91)	0.00-10.00
HLS-EU-Q16^b^	1003	13.66 (2.58)	0.00-16.00
MHL-W-G^c^	1003	51.54 (9.31)	22.00-80.00

^a^PERMA: Positive Emotions, Engagement, Relationships, Meaning, and Accomplishments.

^b^HLS-EU-Q16: European Health Literacy Survey Questionnaire, 16 items.

^c^MHL-W-G: German version of the Mental Health Literacy Tool for the Workplace.

### Bivariate Analyses: Correlates of Well-Being and Health

#### Overview

Detailed descriptive statistics for all groups described below can be found in Table 4. The PERMA well-being score correlated positively with the PERMA health subscale (*r*=0.54, 95% CI 0.49-0.58; *P*<.001).

**Table 4:** Descriptive statistics for the Positive Emotions, Engagement, Relationships, Meaning, and Accomplishments (PERMA) well-being total score and the PERMA health score across the sociodemographic, professional, and health literacy factor levels.

Variable		Well-being, mean (SD)	Health, mean (SD)		
**Gender**					
	Male	7.14 (1.19)	6.57 (1.92)		
	Female	7.20 (1.17)	6.67 (1.91)		
**Age**					
	≤39 years	7.29 (1.06)	6.83 (1.85)		
	40-49 years	6.98 (1.25)	6.50 (1.82)		
	50-59 years	7.20 (1.23)	6.56 (2.09)		
	≥60 years	7.30 (1.20)	6.61 (1.91)		
**Type of school**					
	Primary school	7.12 (1.17)	6.43 (2.04)		
	All other types of school	7.21 (1.18)	6.74 (1.85)		
**Teaching load**					
	<15 lessons	7.10 (1.12)	6.56 (1.95)		
	15-19 lessons	7.06 (1.23)	6.56 (1.95)		
	20-24 lessons	7.10 (1.26)	6.69 (1.93)		
	25-27 lessons	7.27 (1.12)	6.57 (1.89)		
	≥28 lessons	7.32 (1.16)	6.90 (1.88)		
**Years professionally teaching**					
	1-9 years	7.27 (1.11)	6.81 (1.95)		
	10-19 years	7.01 (1.19)	6.41 (1.84)		
	≥20 years	7.29 (1.21)	6.73 (1.95)		
**Health literacy**					
	Inadequate/problematic	6.72 (1.28)	5.97 (2.12)		
	Adequate	7.35 (1.09)	6.88 (1.78)		

#### Sociodemographic Factors

Women did not significantly differ from men concerning their overall well-being (*t*_1000_=–0.70; *P*=.48; *d*=–0.05) or health according to the PERMA model (*t*_1003_=–0.80; *P*=.42; *d*=–0.06).

There were significant differences between the 4 age groups (≤39 years, 40-49 years, 50-59 years, and ≥60 years) regarding overall well-being (*F*_3, 998_=4.25; *P*=.005; η^2^=0.013). On a descriptive level, well-being was lowest among the 40- to 49-year-olds, followed by the 50- to 59-year-olds, the ≤39-year-olds, and the ≥60-year-olds. Post hoc Bonferroni-corrected *t* tests (corrected α=.008) revealed significant differences between the youngest age group and the 40- to 49-year-olds (*t*_510.91_=3.38; *P*<.001; *d*=0.28), but not between the youngest age group and the 50- to 59-year-olds (*t*_625_=1.05; *P*=.30; *d*=0.09) and the ≥60-year-olds (*t*_476_=–0.05; *P*=.96; *d*=–0.01), respectively. With the corrected α-thresholds, the 40- to 49-year-olds did not significantly differ from their peers in the 2 older age groups: 50- to 59-year-olds (*t*_522_=–2.05; *P*=.04; *d*=–0.18) and the ≥60-year-olds (*t*_373_=–2.31; *P*=.02; *d*=–0.26), respectively.

There were no significant differences regarding health when comparing the 4 age groups (*F*_3, 1001_=1.84; *P*=.14; η^2^=0.005).

#### Professional Factors

There were no significant differences in well-being between primary school teachers and teachers who work at other types of schools (*t*_1000_=–1.22; *P*=.22; *d*=–0.08), but primary school teachers reported significantly lower health (according to the PERMA model) than teachers who work at other types of schools (*t*_516.55_=–2.27; *P*=.02; *d*=–0.16).

There were no significant differences in well-being between teachers with different teaching loads (ie, number of lessons per week), *F*_4, 996_=1.79; *P*=.13; η^2^=0.007. Similarly, teaching load was not associated with health (according to the PERMA model), *F*_4, 999_=0.99; *P*=.41; η^2^=0.004.

There was a significant effect of the number of years teaching professionally on well-being (*F*_2, 993_=6.22; *P*=.002; η^2^=0.012). Descriptively, well-being was lowest among those teachers with 10-19 years of professional teaching when compared to those with more or less teaching experience. Post hoc Bonferroni-corrected *t* tests (corrected α=.017) revealed significantly higher well-being among those with 1-9 years of experience than among those with 10-19 years of experience (*t*_652_=2.93; *P*=.004; *d*=0.23). Similarly, well-being was significantly higher among those with 20 or more years of experience than among those with 10-19 years of experience (*t*_678_=–3.12; *P*=.002; *d*=–0.24). There were no significant differences between those with 1-9 years of experience and those with 20 or more years of experience (*t*_657_=–0.25; *P*=.80; *d*=–0.02).

Health (according to the PERMA model) was also significantly associated with the number of years teaching professionally (*F*_2, 996_=3.98; *P*=.02; η^2^=0.008). Descriptively, health was lowest in those teachers with 10-19 years of professional teaching when compared to those with more and less teaching experience. Post hoc pairwise *t* tests with a Bonferroni-corrected α=.017 partly supported this pattern of results by showing significant differences between the teachers with an intermediate level of teaching experience when compared to those with less experience: 1-9 years of experience vs 10-19 years of experience (*t*_654_=2.68; *P*=.008; *d*=0.21). However, there were no differences when comparing those with 20 or more years of experience vs 10-19 years of experience (*t*_681_=–2.17; *P*=.03; *d*=–0.17), and teachers with 1-9 years of experience vs teachers with 20 or more years of experience (*t*_658_=0.54; *P*=.59; *d*=0.04).

#### Health Literacy

When interpreting general health literacy as a dichotomous categorical variable, teachers with inadequate or problematic general health literacy had lower well-being (*t*_409.68_=–7.14; *P*<.001; *d*=–0.55) and lower health (according to the PERMA model; *t*_405.52_=–6.24; *P*<.001; *d*=–0.49) than those with adequate general health literacy.

When looking at health literacy as a continuous measure, there were positive correlations between general health literacy and well-being (*r*=0.26, 95% CI 0.20-0.32; *P*<.001), and general health literacy and health according to the PERMA model (*r*=0.25, 95% CI 0.20-0.31; *P*<.001). Similarly, mental health literacy correlated positively with well-being (*r*=0.20, 95% CI 0.14-0.26; *P*<.001) and health (*r*=0.10, 95% CI 0.04-0.16; *P*=.002), but correlation coefficients were smaller than for general health literacy. All correlation coefficients for the associations of general and mental health literacy with well-being and health were small but statistically significant.

### Multivariate Analyses

#### Regression Assumptions

Preliminary analyses confirmed that relevant assumptions for multiple linear regression were met. Collinearity diagnostics showed no multicollinearity issues in both models, with all variance inflation factors below standard thresholds (<5). Additionally, visual inspection of residual plots supported the assumptions of normality and homoscedasticity, and maximum Cook distances (<1) confirmed the absence of influential outliers.

#### Multivariate Regression Models

In the multivariate regressions predicting PERMA well-being and health, both overall models were statistically significant. The well-being model (*F*_13, 977_=10.66; *P*<.001) explained approximately 11.3% of the variance in the PERMA well-being score (adjusted *R*^2^=0.113). Details for the individual predictors are in Table 5. General health literacy and mental health literacy emerged as significant positive predictors of well-being. Furthermore, the analysis revealed an age- and experience-specific pattern: teachers aged 40-49 years and 50-59 years, as well as those with 10-19 years of teaching experience, reported significantly lower well-being compared to their younger or early-career counterparts. School type, teaching load, and gender did not retain statistical significance in the well-being model. The health model (*F*_13, 980_=8.34; *P*<.001) accounted for 8.8% of the variance in the PERMA health outcome (adjusted *R*^2^=0.088). Details for the individual predictors are in Table 6. While general health literacy was a significant positive predictor, mental health literacy did not reach statistical significance for the health outcome. Health scores were significantly lower for teachers in the midcareer phase (10-19 years) and in both groups aged 50 years and older compared to their younger colleagues (≤39 years). In addition, teaching at nonprimary schools and having a high teaching load (≥28 hours) were positively associated with health.

**Table 5:** Results of the multiple regression analysis evaluating predictors of the Positive Emotions, Engagement, Relationships, Meaning, and Accomplishments (PERMA) well-being scale (n=991).

Predictor			B (SE)^a^		β^b^		*t*		*P* value
Constant			4.76 (0.28)		N/A^c^		17.32		<.001
Gender (reference: male)			0.09 (0.08)		.03		1.05		.30
**Age (reference:** **≤** **39 years)**									
	40-49 years	–0.32 (0.10)		–.12		–3.10		.002	
	50-59 years	–0.28 (0.13)		–.10		–2.14		.03	
	≥60 years	–0.21 (0.16)		–.05		–1.26		.21	
School type (reference: primary)			0.14 (0.09)		.05		1.55		.12
**Teaching load (reference: <15 hours)**									
	15-19 hours	–0.08 (0.14)		–.02		–0.58		.56	
	20-24 hours	–0.02 (0.12)		–.01		–0.15		.88	
	25-27 hours	0.13 (0.12)		.05		1.11		.27	
	≥28 hours	0.27 (0.14)		.08		1.88		.06	
**Years professionally teaching** **(reference: 1-9 years)**									
	10-19 years	–0.21 (0.10)		–.08		–2.10		.04	
	≥20 years	0.09 (0.13)		.04		0.68		.50	
Health literacy (HLS-EU-Q16^d^)			0.11 (0.01)		.23		7.38		<.001
Mental health literacy (MHL-W-G^e^)			0.02 (0.004)		.15		4.68		<.001

^a^B represents unstandardized regression coefficients.

^b^β represents the standardized coefficient.

^c^N/A: not applicable.

^d^HLS-EU-Q16: European Health Literacy Survey Questionnaire, 16 items.

^e^MHL-W-G: German version of the Mental Health Literacy Tool for the Workplace.

**Table 6:** Results of the multiple regression analysis evaluating predictors of the Positive Emotions, Engagement, Relationships, Meaning, and Accomplishment (PERMA) health scale (n=994).

Predictor		B (SE)^a^	β^b^	*t*	*P* value
Constant		3.48 (0.45)	N/A^c^	7.69	<.001
Gender (reference: male)		0.17 (0.14)	.04	1.27	.21
**Age (reference:** **≤** **39 years)**					
	40-49 years	–0.27 (0.17)	–.06	–1.56	.12
	50-59 years	–0.52 (0.21)	–.12	–2.41	.02
	≥60 years	–0.53 (0.27)	–.09	–1.99	.046
School type (reference: primary)		0.44 (0.14)	.10	3.05	.002
**Teaching load (reference: <15 hours)**					
	15-19 hours	–0.12 (0.23)	–.02	–0.50	.61
	20-24 hours	0.09 (0.21)	.02	0.42	.67
	25-27 hours	–0.05 (0.20)	–.01	–0.26	.79
	≥28 hours	0.52 (0.23)	.10	2.26	.02
**Years professionally teaching** **(reference: 1-9 years)**					
	10-19 years	–0.38 (0.17)	–.09	–2.27	.02
	≥20 years	0.21 (0.22)	.05	0.95	.34
Health literacy (HLS-EU-Q16^d^)		0.19 (0.02)	.25	7.87	<.001
Mental health literacy (MHL-W-G^e^)		0.01 (0.01)	.04	1.27	.21

^a^B represents unstandardized regression coefficients.

^b^β=standardized coefficient.

^c^N/A: not applicable.

^d^HLS-EU-Q16: European Health Literacy Survey Questionnaire, 16 items.

^e^MHL-W-G: German version of the Mental Health Literacy Tool for the Workplace.

## Discussion

### Principal Findings

This study investigated the health and well-being of teachers in Germany through the lens of the PERMA model. Regarding our first objective to quantify these constructs, results show relatively high overall health and well-being with some variation across the professional lifespan. Addressing our second objective—exploring the correlates of these outcomes—multivariate analyses revealed that both general and mental health literacy are robust positive predictors of well-being. Notably, we observed a distinct dip in well-being in middle age and midcareer. A similar pattern was observed for the health outcome (without the reincrease in older age). Furthermore, the multivariate model highlighted that lower general (but not mental) health literacy, low teaching loads, and primary school settings were associated with worse health outcomes.

### Well-Being and Health in Teachers From Germany

#### Overview

Our findings add to the literature by showing, in recent data from Germany, that teachers’ well-being, as measured within the PERMA framework, reached moderate to high scores when interpreted relative to the range of the questionnaire. This pattern of results is in line with prior research demonstrating that teachers’ well-being is not generally at risk [10]. In this context, however, it is essential to acknowledge that no validated and widely acknowledged thresholds have been established to categorize individuals into different levels of well-being for this and similar questionnaires, making the interpretation of the absolute scores more difficult [10,37,38]. Compared to the initial validation study of the German-language PERMA-Profiler [37]—which used the same instrument and web-based administration format—our sample reported slightly higher well-being and comparable health. However, because this reference group is a cross-national convenience sample (Austria, Germany, and Switzerland) and data collection took place more than a decade ago, it provides only an approximate benchmark. To offer a more precise, population-specific context, we also cross-referenced our data with a recent study explicitly focusing on the core elements of PERMA well-being among teachers in Germany [15], with which our descriptive findings largely converge.

#### Sociodemographic and Professional Correlates

While no significant associations with gender could be observed in our sample, the results demonstrate that there seems to be a particularly vulnerable period regarding well-being in terms of age: middle-aged teachers reported the lowest well-being. This dip in well-being during midlife is not unique to the group of teachers in Germany, as it has also been found in general population samples from other high-income countries [49-51]. One possible explanation for this finding is that this period is characterized by heightened sensitivity to the negative psychological impact of unrealized life expectations and aspirations [52], which might also be relevant to teachers. In addition, midlife entails unique challenges such as dual caretaking for older parents and adolescent children, being confronted with the onset of physical and cognitive aging, and the pressure to maintain economic stability [53].

When looking at the associations of well-being and health in terms of professional factors, in our sample, years of teaching experience emerged as an important factor for well-being: teachers with an intermediate level of teaching experience (ie, 10-19 years) reported the lowest well-being. A similar pattern was observed for the health outcome, though not fully supported by the corrected pairwise comparisons. Importantly, for the interpretation of these findings, it has to be noted that work experience, measured as the number of years of teaching, is typically correlated with age. Consequently, the underlying mechanisms driving these findings may partly overlap with those associated with age. However, in the multivariate analyses for both outcomes, (intermediate) experience and (middle) age were significant predictors in the absence of multicollinearity (all variance inflation factors <5), which supports the unique contribution of both factors.

Taken together, these findings regarding midlife and midcareer vulnerabilities highlight that teachers in these cohorts might be a priority target group for workplace health and well-being interventions.

Interestingly, workload, operationalized as the teaching load per week, was not significantly associated with either well-being or health in the bivariate analyses. In the multiple regression analysis for the PERMA health outcome, a higher teaching load unexpectedly emerged as a positive predictor of health when controlling for other factors. This is in contrast to prior research that shows associations between working conditions, such as workload and well-being or job satisfaction, which was not mirrored in our findings on the association of well-being and workload [54,55]. This discrepancy might be explained by the fact that the number of lessons taught per week is only an approximation of the actual workload. For example, teaching some subjects might require more work (eg, preparation and grading) outside of the classroom than others. Furthermore, teachers with a lower teaching load may carry significant responsibilities outside of traditional employment, such as caregiving, affecting their well-being and health status. Finally, well-being may be influenced less by workload per se and more by individual psychological and behavioral responses to it. For instance, factors such as effective coping strategies, self-efficacy, and resilience might lower the subjective workload burden for some individuals [11].

Primary school teachers reported significantly lower health (according to the PERMA model) than teachers from other types of schools, both in the bivariate analysis and when all other factors were controlled for in the multiple regression. To date, health-related differences between primary school and other teachers have been observed inconsistently [3,56-58]. A possible explanation for the lower health status in primary school teachers might be that they are confronted with distinct job demands that affect their physical health without necessarily impacting their general well-being, such as voice problems [58].

#### General and Mental Health Literacy

On a descriptive level, health literacy levels of teachers were higher than in the general population, with fewer individuals categorized as having inadequate or problematic health literacy (26.4%) than in recent data from a general population sample (approximately 40% [59]). Similarly, the mean values for the general health literacy questionnaire were higher in teachers than in the general population [34,59]. This might be partially explained by the higher level of education in teachers [60,61]. Importantly, however, it is alarming that about 1 in 4 teachers do not have adequate health literacy, given their important function as facilitators of (health) literacy education in schools [62].

As there are no established thresholds to classify individuals into categories of mental health literacy levels with the instrument used here, we can only compare the descriptive findings to those from other publications. Relative to a general population sample from Germany [34], a similar mean value was observed among teachers in our sample, suggesting that the differences between samples observed for general health literacy do not generalize to mental health literacy. These findings underscore that teachers might benefit from mental health literacy interventions just as much as individuals from the general population. Of note, some interventions have already been developed and evaluated regarding their efficacy in improving aspects of mental health literacy in teachers with positive results [27,63]. For example, a recent randomized controlled trial showed that short educational videos are effective tools for the promotion of mental health literacy among both teachers and students [28].

Consistent with findings from other countries [64,65], we observed significant bivariate associations between lower levels of general and mental health literacy and poorer well-being and health (according to the PERMA model). However, the association between mental health literacy and health did not remain statistically significant when all other factors were controlled for in the regression analysis. Crucially, while the absolute effect sizes were small to moderate [46], they were among the largest observed in this study. Furthermore, in the multivariate analyses, general health literacy emerged as the strongest predictor (yielding the highest beta weights) for both outcomes, followed by mental health literacy for the well-being outcome. This pattern of results underscores the unique associations between health literacies and the 2 outcomes studied here that should be further investigated regarding their practical significance. For example, these findings suggest that health literacy could be a relevant focus for future interventions aimed at supporting teachers’ health and well-being.

### Future Research

Many interventions that already exist to improve teachers’ well-being involve elements of positive psychology [66], in line with the focus on the PERMA model in this investigation. So-called positive psychology interventions are brief interventions developed to enhance well-being with a focus on positive elements such as gratitude, optimism, and kindness, rather than on psychopathology. In the general population, there is evidence for the effectiveness of these interventions on various outcomes [67,68]. In addition, some positive psychology interventions have already been tested among teachers with positive results concerning their efficacy, for example, regarding enhancements in well-being [66]. This area of targeted interventions should be researched more vigorously in the future with the aim of further fostering teachers’ well-being and also exploring potential effects regarding health. Results from this investigation suggest that a particular focus should be placed on midcareer and midlife needs and that the incorporation of health literacy elements might be a promising avenue.

### Limitations

Several limitations have to be considered when interpreting the findings from this study. First, data were collected cross-sectionally at a single point in time. This is a common problem in research on well-being, where correlations with various factors are frequently observed that do not necessarily imply causal relationships [9,69,70]. In addition, some associations are bidirectional in nature. For example, teachers’ well-being might be considered the outcome of job satisfaction, but it can also be seen as its antecedent [9,15]. More in-depth analyses using other methodological approaches, including both longitudinal and interventional studies, are necessary to gain a deeper understanding of these associations. Second, the investigation relied on self-reports in an online survey, which might have led to response biases such as social desirability, recall, or misclassification bias [71,72]. A third limitation concerns the theoretical foundation: the PERMA model, which informed the well-being measure, has been subject to criticism regarding its scientific underpinnings and conceptual clarity [73,74]. At the same time, from an applied and empirical perspective, the model is widely used and has been found to be a helpful tool in the context of understanding well-being, as well as its determinants and malleability, for example, with the help of positive psychological interventions [67,68]. Fourth, the survey achieved a response rate of 61.6% (1005/1631), indicating that a substantial proportion of invited individuals did not participate. Although the data were weighted to mitigate potential bias introduced by the systematic nonresponse of specific population segments, this procedure cannot fully eliminate all disparities. For instance, teachers experiencing poor health or low well-being may have been less motivated to complete the survey. Because the weighting was based on a limited number of sociodemographic factors, the possibility of residual nonresponse bias cannot be entirely ruled out. Fifth, school types were only analyzed regarding differences between primary and all other schools. Collapsing all nonprimary schools into a single category might have obscured some differences. The German educational system encompasses a diverse range of school types across different federal states, making it difficult to create meaningful groupings without compromising statistical power. Future studies with larger subsamples are required to disentangle how specific school types are related to the health and well-being of educators. Lastly, health was measured using a subscale of the PERMA Profiler questionnaire. While the overarching questionnaire is well-validated for its operationalization of well-being, there is a lack of studies explicitly studying the validity of the instrument’s 3-item health scale as an adequate measure of general health. More comprehensive, clinically validated health questionnaires should be used in the future to measure health. In this investigation, a large and positive correlation between the average PERMA health scale value and the response to the single physical health item can be interpreted as an indicator of the scale’s validity.

### Conclusion

In summary, this study provides a comprehensive overview of health and well-being among teachers from Germany using a large and representative sample and thus validates the use of the PERMA framework in this population. Our findings reveal a complex interplay of sociodemographic and professional factors associated with teachers' well-being and health. A key finding is the presence of a vulnerability period for well-being and health in middle-aged teachers and among those with intermediate levels of teaching experience. In addition, the results underscore the potential protective role of general and mental health literacy, with better health literacy levels significantly associated with better health and well-being outcomes across the sample. These competencies could be integrated into standard teacher training as core professional skills, rather than being viewed solely as personal attributes. Targeted health literacy promotion might offer a strategy for mitigating health and well-being problems and improving teachers’ job satisfaction and retention. Moving forward, health promotion efforts in the educational system might benefit from a shift toward tailored support systems, accompanied by ongoing research into their long-term impact on educators and their pupils.

## Data Availability

The datasets generated and analyzed during this study are available from the Stiftung Gesundheitswissen on reasonable request.

## Abbreviations

HLS-EU-Q16: European Health Literacy Survey Questionnaire, 16 items
MHL-W-G: German version of the Mental Health Literacy Tool for the Workplace
PERMA: Positive Emotions, Engagement, Relationships, Meaning, and Accomplishments
